# Serum Levels of 25-Hydroxyvitamin D in Patients with Seborrheic Dermatitis: A Case-Control Study

**DOI:** 10.1155/2021/6623271

**Published:** 2021-02-20

**Authors:** Siavash Rahimi, Negar Nemati, Seyedeh Sareh Shafaei-Tonekaboni

**Affiliations:** Department of Dermatology, Ramsar Campus, Mazandaran University of Medical Sciences, Sari, Iran

## Abstract

Several autoimmune papulosquamous skin conditions such as psoriasis, systemic lupus erythematous, and lichen planus have been associated with vitamin D deficiency or correlated with serum vitamin D level. This study was aimed at comparing the 25-hydroxyvitamin D (25(OH)D) status in patients with facial or scalp seborrheic dermatitis with healthy subjects. This case-control study included 289 patients (118 with psoriasis and 171 sex- and age-matched control subjects) from the outpatient clinic of two hospital dermatology departments in the west of Mazandaran province, Iran. All patients and control subjects were studied during one season to avoid seasonal variations in vitamin D levels. Serum mean ± standard deviation of 25(OH)D levels were signiﬁcantly lower in seborrheic dermatitis patients than in control subjects (20.71 ± 8.16 vs. 23.91 ± 7.78, *P* = 0.007). Serum 25(OH)D levels were negatively associated with the risk of developing seborrheic dermatitis (odds ratio (OR): 0.898, 95% confidence interval (Cl): 0.840–0.960, *P* = 0.002). Also, vitamin D under 30 ng/ml was associated with OR: 4.22 (95% Cl: 1.077–16.534, *P* = 0.039) for seborrheic dermatitis. The severity of scalp disease was significantly associated with serum 25(OH)D level (*P* = 0.003). Cases with severe scalp scores had significantly lower serum 25(OH)D level compared to moderate OR score (*P* = 0.036). A similar trend was not seen in the facial disease. The 25(OH)D values are signiﬁcantly lower in seborrheic dermatitis patients than in healthy subjects. Furthermore, the scalp disease severity was associated with lower serum 25(OH)D level. Our results may suggest that vitamin D may play a role in the pathogenesis of seborrheic dermatitis.

## 1. Introduction

Seborrheic dermatitis is a highly prevalent chronic inflammatory skin condition. Seborrheic dermatitis is characterized by its erythematous papulosquamous morphology with greasy scales in sebaceous gland rich areas, particularly the scalp, face, and body folds. Due to the distinctive facial distribution of the lesions, seborrheic dermatitis has aesthetic consequences and is placed third in dermatological diseases for its potential to impair quality of life [1, 2]. The pathogenesis or associated risk factors of seborrheic dermatitis are currently not fully understood. Earlier, *Malassezia* species were assumed to be the most important pathogenic factor. However, recent investigations have shown that *Malassezia* spp. direct and indirect interaction with the skin immune system is elaborated in the pathogenesis of seborrheic dermatitis [3]. *Malassezia* spp. produce lipases and phosphatases that can degrade sebaceous triglycerides, resulting in an increase in free fatty acids, which may cause activation of toll-like receptor- (TLR-) 2 and induce inflammatory cascade [4]. Moreover, free fatty acids can induce keratinocytes to express thymic stromal lymphopoietin (TSLP), which can induce inflammation with an essential role in directing dendritic cells to stimulate the initiation and progression, a Th2 response [5].

Vitamin D is a secosteroid with a range of biological activities in organs. More than two hundred genes are regulated by vitamin D, either directly or indirectly, which are responsible for many biological activities [6]. Vitamin D receptor (VDR) is expressed by the keratinocytes and 1,25-dihydroxycholecalciferol acting via the VDR, controls proliferation in the basal layer of the epidermis, and subsequently promotes the keratinocytes differentiation as they form the upper layers of the epidermis [7, 8]. Loss of VDR or the capacity to produce 1,25-dihydroxycholecalciferol halts the differentiation of the epidermis and leads to hyperproliferation of the basal layers with the dysfunction of the differentiated layers such as barrier maintenance and immunoregulatory processes [9].

Considering the role of inflammatory cascade in the pathogenesis of seborrheic dermatitis and the inhibitory effect of VDR and vitamin D on immune system, vitamin D deficiency can be suggested as a risk factor for developing seborrheic dermatitis. Furthermore, spontaneous remission of seborrheic dermatitis in summer and variable therapeutic efficacy of topical analogs of vitamin D has been shown previously [10, 11]. However, direct evidence in this regard is lacking. Therefore, this study was aimed at determining the serum level of 25-hydroxyvitamin D (25(OH)D) in patients with clinically manifested facial or scalp seborrheic dermatitis. Moreover, we have also compared seborrheic dermatitis with either facial or scalp involvement.

## 2. Materials and Methods

### 2.1. Patient Selection

This case-control study was conducted on patients who were referred to the general outpatient clinic of Imam Sajjad Hospital, Mazandaran University of Medical Sciences, Ramsar, Iran, in 2019. The study was performed after obtaining the approval of the Ethics Committee of Mazandaran University of Medical Sciences and written informed consent from all participants after explaining the aims and procedures during the study. A single dermatologist visited all patients. Each subject received a careful medical evaluation, including the recording of past personal and family medical history, physical examination, the estimate of time spent outdoors per week, and Fitzpatrick skin type.

One hundred and eighteen patients were enrolled in the case group (*n* = 118). On the other side, one hundred and seventy-one consecutive healthy adults who came to our clinic for cosmetic consultation were enrolled in the control group (*n* = 171). All individuals were from Ramsar and Tonekabon city to avoid geographic alterations in ultraviolet exposure and vitamin D levels. Patients were studied during the same period (autumn 2019) to evade seasonal differences in vitamin D levels.

The exclusion criteria were patients with malabsorption, bone disease, autoimmune disease, chronic renal failure, liver disease, cancer, and pregnancy. They were also excluded if they used anticonvulsants, phototherapy, immunosuppressive, contraceptive, topical treatment for seborrheic dermatitis, calcium, multi-vitamins, vitamin D, or vitamin D/mineral fortified food during the past three months. The diagnosis of seborrheic dermatitis was based on clinical ﬁndings. Inclusion criteria were age between 18 and 65 years and diagnosis of seborrheic dermatitis.

### 2.2. Dermatitis Area and Severity Index (SEDASI) Score for Facial Seborrheic Dermatitis

Clinical severity of facial seborrheic dermatitis was evaluated using Seborrheic SEDASI score [12]. This severity scoring system was created based on the evaluation of four facial regions, each demonstrating nearly a quarter of the entire facial surface: forehead (containing upper eyelids and eyebrows), nose (containing nasolabial folds), right cheek (containing ear, lower eyelids, and chin), and left cheek (containing ear, lower eyelids, and chin). For each region, four items were measured: (1) erythema degree (none = 0; pink = 1; pink-red = 2; intense red = 3); (2) extension of seborrheic dermatitis lesions expressed as percentage area of involvement (no visible lesions = 0; 1–9% = 1; 10–29% = 2; 30–49% = 3; 50–69% = 4; 70–89% = 5; 90–100% = 6); (3) presentation pattern (no visible lesions = 0; few small scattered patches = 1; multiple and/or clustered patches = 2; large and/or confluent patches = 3); (4) scaling degree (none = 0; tiny scales = 1; medium-size thicker scales = 2; large, thickened scales = 3). Total score may range from 0 (no seborrheic dermatitis) to 60 (seborrheic dermatitis of the worst possible degree).

### 2.3. Clinical Severity Score Criteria (CSSC) for Seborrheic Dermatitis of the Scalp

Clinical severity of seborrheic dermatitis in the scalp was measured using Shin et al. method [13]. The severity was evaluated by the dermatologist's evaluation of dandruff, erythema, and lesional extent with a 4-point scale score. The clinical severity score is the sum of three scores.

### 2.4. Serum 25(OH)D Measurement

A 5 mL blood sample was taken from each patient. They were stored at −20°C, and 25(OH)D was performed on the same day and under similar conditions. The serum level of 25(OH)D was measured using a commercially available enzyme-linked immunosorbent assay kit (Minneapolis, USA). Vitamin D insufficiency is defined as a 25(OH)D between 30 and 20 ng/ml and vitamin D deficiency as a 25(OH)D <20 ng/ml [14].

### 2.5. Statistical Analyses

Statistical analyses were performed using the SPSS software version 25.0 for Windows (SPSS Inc., Chicago, IL). The Levene and Kolmogorov–Smirnov test were used to examine the equality of variances and distribution of variables, respectively. In case of a normal distribution, a Student *t*-test was applied to compare mean values of quantitative variables, and when not normal, the Mann–Whitney *U* test was used. Qualitative variables were analyzed with Pearson's chi-square test or with Fisher exact test if at least one cell had an expected count of less than five or had more than four cells. Binary or multinomial logistic regression models were used to calculate the odds ratio (OR) and to measure the association between seborrheic dermatitis and different quantitative and qualitative variables in a univariate or multivariate analysis. Correlations among variables were studied by Pearson coefficient or Spearman's correlation coefficient depending on the normality of the variable. Linear regression analysis was used to determine the SEDASI or CSSC as an independent predictor of 25(OH)D levels. For comparison of 25(OH)D and CSSC, one-way ANOVA with Welch's F-test was used, and post hoc analysis was performed with the Games–Howell test. Quantitative and qualitative variables are expressed as mean ± standard deviation (SD) and percentages (numbers), respectively. A *P* value of less than .05 was considered statistically significant.

## 3. Results

### 3.1. Clinical Characteristics of Subjects

In this study, two hundred and eighty-nine patients were evaluated. The mean ± SD age of participants in the case and control groups were 42.76 ± 15.51 and 44.96 ± 15.15, respectively (*P* = 0.18). 47.4% (56) of participants in the case group and 54.4% (62) in the control group were female (*P* = 0.247). Although participants' BMI in the control group was higher than the case group, this difference was not statistically significant (25.7 ± 3.2 vs. 26.57 ± 4.05, *P* = 0.077). In the case group, 22.9% (27), 59.3% (70), and 17.8% (21) had type II, III, and IV Fitzpatrick skin type. On the other hand, 29.8% (51), 56.3% (94), and 15.2% (26) of participants in the control group had type II, III, and IV Fitzpatrick skin types, respectively. The difference in the Fitzpatrick skin type among groups was not statistically significant (*P* = 0.413). Mean ± SD of estimated time spent outdoors expressed as hours per week in case and control groups were 17.07 ± 12.02 and 15.27 ± 11.28, respectively (*P* = 0.196). Among the case group, 60 patients had only facial involvement, and 52 patients had scalp seborrheic dermatitis. Also, six patients had both facial and scalp seborrheic dermatitis. Mean ± SD of SEDASI and CSSC scores were 6.06 ± 3.4 and 2.83 ± 1.74, respectively. Mean ± SD SEDASI score in sufficient, insufficient, and deficient 25(OH)D status was 5 ± 1.88, 6.64 ± 3.86, and 6 ± 3.43, respectively (*P* = 0.453). CSSC scores based on sufficient, insufficient, and deficient 25(OH)D levels were 2.6 ± 1.07, 2.73 ± 1.7, and 4.5 ± 2.89, respectively (*P* = 0.134).

### 3.2. Association of 25(OH)D with Seborrheic Dermatitis

25(OH)D was significantly lower in the case group compared to the control group (20.71 ± 8.16 vs. 23.91 ± 7.78, *P* = 0.007). The frequency of sufficient, insufficient, and deficient status of serum 25(OH)D in the facial seborrheic dermatitis subgroup was 8 (12.9%), 20 (32.3%), and 34 (54.8%), respectively. This frequency among the scalp seborrheic dermatitis subgroup was 16 (28.6%), 22 (39.3%), and 18 (32.1%), respectively. The frequency of these three 25(OH)D status in the healthy group was 39 (22.8%), 79 (46.2%), and 53 (31%), respectively. Vitamin D insufficiency was more prevalent among healthy individuals, and vitamin D deficiency was more prevalent in cases. This difference was statistically significant among groups (*P* = 0.013). Subgroup analysis showed that patients with facial seborrheic dermatitis had significantly lower serum 25(OH)D compared to the healthy group (19.23 ± 7.97 vs. 23.91 ± 7.78, *P* < 0.001). However, serum 25(OH)D of patients with scalp seborrheic dermatitis were not significantly different with either facial seborrheic dermatitis group or healthy group (22.34 ± 8.13, *P* = 0.096 and 0.416, respectively). The binary logistic regression model of the seborrheic dermatitis group showed a significant difference between vitamin D status (deficient, sufficient, or under 30 ng/ml) and serum 25(OH)D level (Table 1).

### 3.3. Correlation between Variables and 25(OH)D

Evaluating the correlation between quantitative variables showed a weak positive correlation between age, BMI, and 25(OH)D (BMI: *r* = 0.246, *P* < 0.01, 25(OH)D: *r* = 0.261, *P* < 0.05). Also, a strong correlation was found between SEDASI and CSSC (*r* = 0.961, *P* = 0.002). However, no significant correlation was found between SEDASI or CSSC and 25(OH)D (*r* = −0.049, *r* = −0.015, *P* > 0.05, respectively). The linear regression model also did not show any significant association between SEDASI or CSSC and 25(OH)D (SEDASI : *R*^2^ = 0.024, *P* = 0.219, CSSC : *R*^2^ = 0.021, *P* = 0.279).

### 3.4. Association of Disease Severity with Serum 25(OH)D

**Figure 1:**
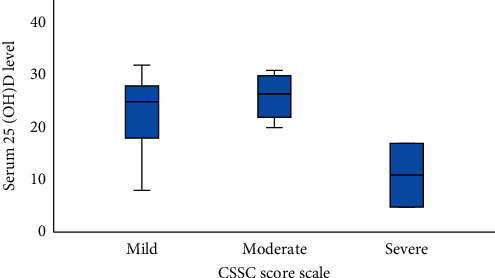
Serum 25(OH)D level based on the CSSC score scale. Data are shown as mean, minimum, maximum, and SD.

CSSC was significantly associated with serum 25(OH)D level (*P* = 0.003) (Figure 1). Cases with severe CSSC scores had significantly lower serum 25(OH)D levels compared to moderate CSSC scores (*P* = 0.036). However, the difference in serum levels of 25(OH)D remained insignificant between moderate and mild or mild and severe groups (*P* = .116 and .085, respectively). Although serum 25(OH)D was lower in patients with moderate SEDASI score, the difference was insignificant (11.9 ± 8.19 vs. 19.62 ± 8.24, *P* = 0.095).

Categorical analysis of 25(OH)D status with SEDASI or CSSC also showed a significant association of 25(OH)D status and CSSC (*P* = 0.003) but not with SEDASI (*P* = 0.267).

## 4. Discussion

To the best of our knowledge, this is the first study to assess vitamin D status in patients with seborrheic dermatitis. There were significant differences in the mean 25(OH)D concentration between patients with seborrheic dermatitis and healthy individuals.

Although no study has evaluated the association of vitamin D deficiency and seborrheic dermatitis, several studies have evaluated the efficacy of topical analogs of vitamin D in seborrheic dermatitis. Early uncontrolled and small studies showed encouraging results for the treatment of seborrheic dermatitis with topical analogs of vitamin D [15–17]. Nonetheless, later studies conducted on larger patient groups appear to contradict these results. Berth-Jones and Adnitt, in a double-blind, placebo-controlled, multicenter randomized clinical trial, showed that calcipotriol cream (50 *µ*g/mL) was inferior to the vehicle after four weeks in facial seborrheic dermatitis [18]. Basak and Ergin also showed that calcipotriol (50 *µ*g/mL) solution was inferior to betamethasone valerate (1 mg/mL) solution for scalp seborrheic dermatitis after four weeks of treatment [19]. In the most recent study by Yap, a combination of calcipotriol plus betamethasone dipropionate gel was used in the treatment of moderate to severe scalp seborrheic dermatitis. This study showed that the combination of betamethasone and calcipotriol is useful in the treatment of moderate to severe scalp disease. However, the study was quasi-experimental and retrospective [20]. The observed discrepancy between studies can be due to variable experimental design, unknown status of basal serum 25(OH)D level, and disease severity. Topical or oral vitamin D analogs can act differently based on disease severity, type of disease, and basal 25(OH)D levels.

Our study has shown that serum vitamin D is significantly lower in patients with seborrheic dermatitis compared to the control group. Moreover, we divided the case group based on the type of seborrheic dermatitis. It was shown that patients with facial involvement had significantly lower vitamin D compared to patients with scalp disease or control group. However, the mean serum level of vitamin D was not significantly different between seborrheic dermatitis patients with scalp involvement. Categorizing subjects based on their vitamin D level revealed the same results. Binary logistic regression showed that vitamin D insufficiency (<30 ng/ml) or vitamin D deficiency (<20 ng/ml) is associated with a higher risk of developing seborrheic dermatitis (OR: 4.22 and 1.845, respectively). Vitamin D insufficiency alone was not associated with the development of seborrheic dermatitis. Quantitative analysis of serum level of 25(OH)D also showed a significant association with the development of 25(OH)D (OR: 0.898). The association of the severity of seborrheic dermatitis and 25(OH)D was also evaluated in this study. It was shown that the severity of scalp disease is significantly correlated with 25(OH)D. patients with severe scalp disease had significantly lower serum 25(OH)D level compared to moderate CSSC scores. Nevertheless, the difference in serum levels of 25(OH)D remained insignificant between moderate and mild or mild and severe groups.

Inadequate 25(OH)D levels in patients with seborrheic dermatitis may be related to isoenzymes alterations involved in the vitamin D metabolism. The isoenzyme polymorphism has also been shown to influence serum 25(OH)D levels [9]. Some studies have shown isoenzyme polymorphisms among patients with inflammatory skin disease such as atopic dermatitis [21]. However, no similar study has been conducted for seborrheic dermatitis. Also, studies have failed to show any sign of systemic inflammation in patients with seborrheic dermatitis; therefore, deficiency in 25(OH)D level is not secondary to inflammation [22].

Although the present study has suggested the association of vitamin D deficiency in patients with seborrheic dermatitis, several limitations are posed. In this study, participants were asked to estimate their weekly time spent outdoors. Even though it is not an ideal method to estimate sun exposure, the same method was applied in both groups, and no signiﬁcant differences were found. Confounding factors associated with ultraviolet radiation, such as changes in latitude, attitude, or climate, were minimized by recruiting all patients from the same geographic location and studying them all in the same period (autumn season). Finally, further studies with a larger population of patients with different types of diseases from various geographical locations are required to elucidate the causative role of vitamin D deficiency in seborrheic dermatitis.

## 5. Conclusion

To the best of our knowledge, this is the first study to assess vitamin D status in patients with seborrheic dermatitis. Serum 25(OH)D levels are signiﬁcantly lower in seborrheic dermatitis patients than in healthy control subjects. These data support the notion of the causative role of vitamin D deficiency in the development of seborrheic dermatitis. Also, subgroup analysis showed that facial seborrheic dermatitis is associated with lower serum vitamin D but not scalp disease. In scalp seborrheic dermatitis, low 25(OH)D levels are associated with higher severity of the disease.

## Figures and Tables

**Table 1:** Binary logistic regression model for seborrheic dermatitis risk.

Variable	OR	95% Cl	*p* value
Gender (male vs. female)	1.26	0.773–2.061	0.351
BMI	0.942	0.88–1.008	0.084
Serum 25(OH)D level	0.898	0.840–0.960	**0.002**
Vitamin D insufficiency or deficiency (<30 ng/ml)	4.22	1.077–16.534	**0.039**
Vitamin D, sufficient (≥30 ng/ml)	0.854	0.783–1.007	0.07
Vitamin D, insufficient (<30 and >20 ng/ml)	1.594	0.844–3.013	0.151
Vitamin D, deficient (<20 ng/ml)	1.845	1.081–3.152	**0.025**
OR, odds ratio; CI, confidence interval			

## Data Availability

The data used to support the findings of this study are available from the corresponding author upon request.
